# Pregnant Smokers Receiving Opioid Agonist Therapy Have an Elevated Nicotine Metabolite Ratio: A Replication Study

**DOI:** 10.1093/ntr/ntaa066

**Published:** 2020-04-18

**Authors:** Henry R Kranzler, Yukiko Washio, Leah R Zindel, Kevin G Lynch, Dennis Hand, Rachel F Tyndale, Cheryl Oncken, Robert Schnoll

**Affiliations:** 1 Center for Studies of Addiction, University of Pennsylvania Perelman School of Medicine, Philadelphia, PA; 2 Mental Illness Research, Education and Clinical Center, Crescenz Veterans Affairs Medical Center, Philadelphia, PA; 3 Substance Use, Gender and Applied Research, RTI International, Research Triangle Park, NC; 4 Departments of Obstetrics & Gynecology and Psychiatry & Human Behavior, Thomas Jefferson University, Philadelphia, PA; 5 Campbell Family Mental Health Research Institute, Centre for Addiction and Mental Health, University of Toronto, Toronto, ON, Canada; 6 Departments of Pharmacology & Toxicology and Psychiatry, University of Toronto, Toronto, ON, Canada; 7 Departments of Medicine and Obstetrics and Gynecology, University of Connecticut School of Medicine, Farmington, CT; 8 Center for Interdisciplinary Research on Nicotine Addiction, University of Pennsylvania Perelman School of Medicine, Philadelphia, PA

## Abstract

**Introduction:**

Pregnant women exposed chronically to opioids smoked more cigarettes per day (CPD) and had a higher nicotine metabolite ratio (NMR), 3-hydroxycotinine/cotinine, a biomarker of nicotine metabolism and clearance, than those not receiving opioids. We examined CPD and NMR in a group of pregnant smokers, a quarter of whom were receiving opioid agonist therapy (OAT).

**Aims and Methods:**

Pregnant smokers recruited to participate in a placebo-controlled trial of bupropion for smoking cessation provided a blood sample for measurement of NMR.

**Results:**

Half (52.4%) of the 124 women with NMR data were African American. OAT-treated women (*n* = 34, 27.4%; 27 receiving methadone and 7 buprenorphine) were more likely to be white (79% vs. 30%, *p* < .001) and to have a lower mean PHQ-9 total score (2.91 [*SD* = 2.83] vs. 4.83 [*SD* = 3.82], *p* = .007). OAT-treated women reported smoking more CPD (9.50 [*SD* = 5.26] vs. 7.20 [*SD* = 3.65], *p* = .005) and had higher NMR (0.78 [*SD* = 0.36] vs. 0.56 [*SD* = 0.25], *p* = .001) than the non-OAT-treated group. In a linear regression analysis adjusting for race, depression severity, and CPD, NMR was greater in the OAT group (*p* = .025), among whom the daily methadone-equivalent dosage correlated with NMR (Spearman’s *ρ* = 0.49, *p* = .003).

**Conclusions:**

Consistent with the findings of Oncken et al. (2019), we found that OAT smokers smoked more and had higher NMR than non-OAT smokers. As higher NMR is associated with a reduced likelihood of smoking cessation, the effects on NMR of both pregnancy and OAT could contribute to a lower smoking cessation rate in pregnant smokers receiving chronic opioid therapy.

**Implications:**

We replicated the finding that the NMR is significantly greater among pregnant smokers receiving OAT than those not receiving this treatment for opioid use disorder. Furthermore, we found that the dosage of the OAT was significantly associated with the NMR level. These findings may contribute to a poorer response to smoking cessation treatment in pregnant women treated with OAT, particularly those receiving high-dose therapy, and raise the question of whether novel approaches are needed to treat smoking in this subgroup of pregnant smokers.

## Introduction

In 2016, 9.4% of U.S. women reported smoking before becoming pregnant, with rates decreasing during pregnancy.^1^ Nonetheless, cigarette smoking during pregnancy is estimated to contribute to 5.3%–7.7% of preterm deliveries, 13.1%–19.0% of low-birth-weight deliveries, 23.2%–33.6% of cases of sudden infant death syndrome, and 5.0%–7.3% of preterm-related deaths.^2^ When treated with FDA-approved medications for tobacco use, pregnant smokers show lower rates of smoking cessation than nonpregnant women.^3^ This is due, in part, to the induction of the enzymes CYP2A6 and UGT2B10 and a higher rate of nicotine metabolism, which begins by 12 weeks of gestation and continues throughout pregnancy.^4,5^

The ratio of the nicotine metabolites 3′-hydroxycotinine (3HC) to cotinine (nicotine metabolite ratio [NMR]) reflects the rate of nicotine metabolism and clearance. The NMR is largely determined by the activity of CYP2A6, encoded by the gene *CYP2A6*, and other genetic and environmental influences.^6,7^ The NMR is highly reproducible and independent of the time since the last cigarette.^8^ Importantly, slow metabolizers of nicotine have significantly higher quit rates than faster metabolizers, both in the absence of pharmacotherapy and with nicotine replacement therapy and in smokers of both European^9^ and African ancestry.^10^

Opioid use disorder (OUD) is also an important factor contributing to the effectiveness of smoking cessation efforts. For example, methadone-maintained OUD patients have a smoking prevalence that is about fourfold and sustained smoking cessation rates with treatment half or less that of the general population.^11^ As in the general population, opioid use in pregnancy has increased dramatically in recent years.^12^ Although opioid agonist therapy (OAT) is the recommended treatment for OUD during pregnancy, chronic opioid exposure appears to augment the effects of pregnancy on NMR.^13^ Thus, efforts to promote smoking cessation during pregnancy among women receiving OAT may be less successful than among pregnant smokers not receiving OAT. In the present study, we examined the daily smoking rate and NMR in pregnant smokers differentiated by the presence or absence of OAT.

## Methods

### Participants

Subjects (*N* = 129) were recruited to participate in a randomized, placebo-controlled trial of bupropion for smoking cessation among pregnant smokers (clinicaltrials.gov: NCT02188459). They were eligible to participate if they smoked at least 3 cigarettes per day (CPD) for the preceding 7 days, wanted to quit smoking, were at 13–26 weeks of gestation, at least 18 years old, able to speak English, and able to sign written informed consent and commit to completing the study procedures. We excluded individuals with a current substance use disorder other than nicotine and opioid use disorders, including alcohol use disorder, multiple gestations other than twins, an unstable psychiatric or medical problem, current use of psychotropic medication, a known congenital abnormality, a history of a seizure disorder, current use of any other smoking cessation medication, anorexia or bulimia, or use of other tobacco products. The study was approved by institutional review boards at all participating centers and was conducted from October 30, 2014 through January 22, 2020.

The current analyses include 124 pregnant smokers with biomarker data at baseline. We excluded five participants whose NMR could not be calculated, either because a blood sample could not be obtained (*n* = 1) or their cotinine and 3HC levels were below the level of quantification (*n* = 4).

### Procedure and Measures

Baseline demographic and clinical variables included age, race, marital status, gestational age (in weeks), and depression symptom severity (measured using the nine-item Patient Health Questionnaire or PHQ-9^14^). We obtained a medication history and OAT was identified through self-report and medical record review. At baseline, the average number of cigarettes smoked per day in the past 7 days was assessed by self-report, and the Fagerström Test for Cigarette Dependence (FTCD) was administered.^15^

### Analytic Chemistry

Serum samples were assayed for the concentration of nicotine, cotinine, and trans-3HC by Liquid Chromatography with tandem mass spectrometry as previously described^8^ with limits of quantification of 1.0 ng/mL whole blood for each compound. We excluded individuals with cotinine less than 10 ng/mL, which is suggestive of non-daily smoking^16^ and yields unstable NMR measurements because cotinine is not at a steady state.

### Data Analysis

NMR was calculated as the ratio of 3HC/cotinine. In bivariate analyses, we tested differences in baseline characteristics by OAT status using Wilcoxon tests for continuous variables and chi-square tests for categorical variables. The distribution of NMR showed low levels of skewness in both the OAT (skew = 0.21) and non-OAT (skew = 0.24) groups, with no outliers, so we analyzed the raw NMR score. We conducted multivariable linear regression to determine the associations between NMR and OAT status, with CPD, PHQ-9 total, and race included as covariates, as they differed significantly by OAT status. For the regression analysis, we report the unstandardized regression coefficients, corresponding to the difference in NMR mean between the OAT and non-OAT groups, adjusting for the covariates. Because the distribution of OAT users differed by race, in a secondary regression analysis, we examined the interaction of race with OAT status on NMR.

Additionally, within the OAT group, we examined the associations between OAT daily dose and NMR using the daily dose reported by participants (in milligrams). To establish equivalence between dosing of methadone and buprenorphine, we multiplied the buprenorphine daily doses by 5.^17^ To accommodate an outlier on daily dose in the methadone group, we used Spearman rank correlations to summarize the association between NMR and daily dose level within each group, with a robust regression method (using a bisquare weight function) to test for main effects of daily dose and interactions with OAT type.

We performed all analyses using SAS/STAT software, version 9.4 (SAS Institute Inc., Cary, NC).

## Results

Table 1 shows the baseline demographic and clinical characteristics of the 124 pregnant smokers included in the analyses comparing the OAT and non-OAT groups. The groups were similar on mean age (29.91 [*SD* = 5.29] and 27.96 [*SD* = 4.63] years in the OAT and non-OAT groups, respectively) and the likelihood of ever having married (41% vs. 38%, respectively). However, the racial composition of the groups differed significantly, with the OAT group predominantly (79%) of European ancestry and the non-OAT group predominantly (64%) of African ancestry. The non-OAT group had a higher mean PHQ-9 total score (4.83 [*SD* = 3.82]) than the OAT group (2.91 [*SD* = 2.83]). The mean number of CPD was significantly higher in the OAT group (9.50 [*SD* = 5.26]) than the non-OAT group (7.20 [*SD* = 3.65]), although the FTCD total score was not (4.15 [*SD* = 1.99] in the OAT group and 3.71 [*SD* = 1.80] in the non-OAT group). The gestational age did not differ by group, which obviated controlling for this potential effect on NMR.^5^ Most patients in the OAT group were being treated with methadone (*n* = 27 or 79.4%; mean daily dosage = 91.0 mg [*SD* = 62.6]) with the remainder receiving buprenorphine (*n* = 7 or 20.6%; mean daily dosage = 14.3 mg [*SD* = 3.2]). One participant reported a confirmed daily methadone dose of 360 mg; for the other 26 participants, the mean daily dose was 80.62 mg (*SD* = 32.70).

**Table 1.** Pretreatment Demographic and Clinical Variables by Opioid Agonist Therapy (OAT) Status (*N* = 124)

	OAT status		
	Non-OAT	OAT	*p* ^a^
Age in years, mean (*SD*)	28.0 (4.6)	29.9 (5.3)	.087
Race, *n* (%)			**<.0001**
AA/black	58 (64%)	7 (21%)	—
EA/white	27 (30%)	27 (79%)	—
Other	5 (6%)	0 (0%)	—
Marital status, *n* (%)			.73
Never married	56 (62%)	20 (59%)	—
Ever married	34 (38%)	14 (41%)	—
Gestational age in weeks, mean (*SD*)	21.1 (3.9)	20.6 (4.6)	.78
PHQ-9 total score, mean (*SD*)	4.8 (3.8)	2.9 (2.8)	**.007**
Cigarettes/day, mean (*SD*)	7.2 (3.6)	9.5 (5.3)	**.005**
FTCD total score, mean (*SD*)	3.7 (1.8)	4.1 (2.0)	.36
Serum cotinine (ng/mL), mean (*SD*)	111.1 (70.9)	105.2 (61.7)	.87
Serum 3HC (ng/mL), mean (*SD*)	57.5 (44.2)	73.6 (50.4)	.074
Nicotine metabolite ratio, mean (*SD*)	0.6 (0.3)	0.8 (0.4)	**.001**

*SD* = standard deviation; AA = African American; EA = European American; FTCD = Fagerström Test for Cigarette Dependence; 3HC = 3-hydroxycotinine; PHQ-9 = Patient Health Questionnaire-9. Numbers in bold are statistically significant.

^a^ *p*-values calculated using *F*-test for continuous variables and chi-square statistic for categorical variables.

### Nicotine Metabolite Concentrations and Ratio

Blood cotinine concentration did not differ by OAT status (105.16 ng [*SD* = 61.70] and 111.13 ng [*SD* = 70.89] in the OAT and non-OAT groups, respectively), with a nonsignificant trend (*p* = .074) for a greater concentration of 3HC in the OAT group (73.62 ng/mL [*SD* = 50.39]) than the non-OAT group (57.54 ng/mL [*SD* = 44.18]). The mean NMR was significantly greater in the OAT group (0.78 [*SD* = 0.25]) than the non-OAT group (0.56 [*SD* = 0.25]), though within the OAT group, the mean NMR did not differ between methadone-treated women (0.78 [*SD* = 0.40]) and the buprenorphine-treated women (0.76 [*SD* = 0.12], *p* = .98).

### Predictors of NMR

The results of the linear regression model of NMR adjusted for race, PHQ-9 score, and CPD are presented in Table 2. The OAT group had a higher NMR than the non-OAT group (*b* = 0.14, *SE* = 0.06, *p* = .025), with African American (AA) women having a lower NMR than European American (EA) women (*b* = −0.15, *SE* = 0.05, *p* = .008). We conducted a secondary analysis that was restricted to the 65 AAs and 54 EAs (which exclude five individuals of “other” race), which yielded results very similar to those in the full sample: the expected NMR value among the OAT is 0.15 (*SE* = 0.06) larger than in OAT nonusers (*p* = .016). Within this reduced sample of 118, there are 7 AAs (10.8%) and 27 EAs (50.0%) who were receiving OAT, a distribution within populations that yields lower power than a more even distribution would. An analysis of the interaction of race by OAT was not significant: *χ*^2^^1^ = 0.59, *p* = .44. Within AAs, there is a small, nonsignificant effect: the expected NMR value among OAT users is 0.08 (*SE* = 0.11) larger than in OAT nonusers (*p* = .47). Within EAs, the expected NMR value among OAT users is 0.18 (*SE* = 0.08, *p* = .015) larger than in OAT nonusers, results that were similar to those in the full sample.

**Table 2.** Multivariable Linear Regression Model of Nicotine Metabolite Ratio as a Function of Opioid Agonist Therapy Status, Depression Severity, Race, and Smoking Intensity (*N* = 124)^a^

	*β* coefficient^b^	*SE*	*p*
Opioid agonist therapy	0.14	0.06	.025
Cigarettes per day (CPD)	−0.0006	0.006	.920
PHQ-9 score	−0.006	0.007	.373
Race/ethnicity (vs. white)			
African American	−0.15	0.05	.008
Other/Refused	−0.02	0.13	.859

*SE* = standard error.

^a^Predictors: opioid agonist therapy and CPD; covariates: Patient Health Questionnaire-9 (PHQ-9) and race.

^b^We report the unstandardized regression coefficient.

Neither CPD nor PHQ-9 score was associated with NMR. Although changes in NMR have been observed across pregnancy,^5^ we tested women at approximately the same gestational age.

Among women receiving OAT (*n* = 34), a higher OAT dose was associated with higher NMR levels (Spearman’s *ρ* = 0.49, *p* = .003). The associations were of a similar magnitude within the methadone-only (*n* = 27, *ρ* = 0.50, *p* = .05) and the buprenorphine-only (*n* = 7, *ρ* = 0.45, *p* = .31) subgroups. In the main-effect robust regression model, there was a significant positive association (*β* = 0.002, *SE* = 0.001, *p* = .044) between daily OAT dose and NMR, but no interaction of dose × OAT subgroup (*p* = .95).

## Discussion

After controlling for relevant covariates, we found that the use of OAT was associated with higher NMR among pregnant smokers. Specifically, although cotinine levels were similar among smokers irrespective of OAT, 3HC levels were marginally higher and NMR was significantly higher among OAT smokers, findings that are consistent with those of Oncken et al.^13^ In contrast to the prior report, however, in which the NMR value was nominally greater among women treated with methadone (56% of the sample; median NMR = 0.63) than among those receiving buprenorphine (32% of the sample; median NMR = 0.55), the two OAT medication groups in our sample were nearly identical on NMR (methadone: 79.4% of the sample; NMR = 0.78; buprenorphine 21.6%; NMR = 0.76). We also found a moderate (*ρ* = 0.49), but significant, correlation between the dosage of OAT and NMR, consistent with a pharmacological effect of OAT on NMR.

We found a nominally larger effect of OAT on NMR in EAs than AAs. However, because of the small number of AAs who were receiving OAT (*n* = 7), we cannot draw a conclusion as to whether race affects OAT’s impact to increase NMR during pregnancy. Future studies in larger samples of AAs who are receiving OAT are needed to evaluate the racial difference.

The prevalence and persistence of cigarette smoking in individuals with OUD far exceed that of the general U.S. adult population^18^ and the severity of nicotine dependence is significantly greater than that among smokers without OUD.^19^ As reviewed above, a more rapid nicotine metabolism, reflected by a higher NMR, is associated with a lower likelihood of smoking cessation. Thus, in addition to the increase in NMR associated with pregnancy, chronic opioid exposure also appears to increase NMR, and thus likely increases nicotine metabolism. These may jointly contribute to the poorer response to smoking cessation treatment seen among pregnant women with OUD.

The mechanism by which OAT increases the NMR during pregnancy remains to be determined. It could be via induced CYP2A6, but opioids also compete in a dose-dependent manner for 5′-diphospho-glucuronosyltransferase (UGT) enzymes,^20^ which metabolize 3HC to 3HC glucuronide for excretion. Chronic opioid exposure is associated with lower 3HC metabolism, which increases NMR, an effect that is consistent with the correlation observed here between OAT dose and NMR.

Oncken et al.^13^ found a significant association of opioid exposure and NMR only for methadone. In our sample, the NMR values for the methadone- and buprenorphine-treated groups were similar. Given the small number of buprenorphine-treated women in both studies, further research on the effects of chronic opioid exposure on NMR in pregnant smokers is warranted in samples large enough to compare the effects of different opioids.

We found a moderate correlation between OAT dosage and NMR, which was comparable in magnitude in the methadone and buprenorphine groups. This contrasts with a study of nonpregnant smokers maintained on buprenorphine, in which there was no effect of the drug’s dosage on NMR.^21^ Our study was limited by the small number of smokers receiving OAT. Furthermore, although we controlled for a variety of potential confounders, we did not have information on whether the participants smoked menthol cigarettes, which can affect nicotine metabolism; Oncken et al.^13^ found no such effect of menthol cigarettes. Further research is needed to determine whether a dose–response relationship that appears to exist for OAT differs with the specific maintenance medication and the impact of menthol cigarettes on this relationship.

In summary, we replicated the reported finding that pregnant smokers receiving chronic opioid therapy have a higher NMR than pregnant smokers not receiving opioids.^13^ The moderate correlation that we observed between the dosage of OAT and NMR is consistent with a pharmacokinetic interaction between nicotine and chronic opioid therapy. Further research is needed to replicate these findings in a larger sample of pregnant smokers receiving OAT, so as to ascertain whether there are different effects among opioid drugs, to identify other factors that potentially affect NMR, and to elucidate the mechanisms by which these effects occur.

## Supplementary Material

A Contributorship Form detailing each author’s specific involvement with this content, as well as any supplementary data, are available online at https://academic.oup.com/ntr.

ntaa066_suppl_Supplementary_Taxonomy_Form

## Funding

This study was supported by the National Cancer Institute (R01 CA184315). RFT’s role was supported, in part, by funding from the Canada Research Chairs program (Canada Research Chair in Pharmacogenomics), Canadian Institutes of Health Research (Foundation grant FDN-154294), and the Centre for Addiction and Mental Health.

## Declaration of Interests

HRK is a member of the American Society of Clinical Psychopharmacology’s Alcohol Clinical Trials Initiative, which was supported in the last 3 years by AbbVie, Alkermes, Ethypharm, Indivior, Lilly, Lundbeck, Otsuka, Pfizer, Arbor, and Amygdala Neurosciences and is named as an inventor on PCT patent application #15/878,640 entitled: “Genotype-guided dosing of opioid agonists,” filed January 24, 2018. RS has received medication and placebo free from Pfizer and has provided consultation to Pfizer, GlaxoSmithKline, and CuraLeaf. RFT has consulted for Quinn Emanuel and Ethismos Research, Inc.; sits on scientific advisory boards (with no financial compensation), for example, Health Canada’s Vaping SAB; and received research support from Global Research Awards for Nicotine Dependence (GRAND), an independently reviewed competitive grants program supported by Pfizer, to the University of Toronto.
