# Supplementary material for: Pregnant Smokers Receiving Opioid Agonist Therapy Have an Elevated Nicotine Metabolite Ratio: A Replication Study
Source: Nicotine Tob Res. 2020 Apr 18;22(10):1923–7. doi: 10.1093/ntr/ntaa066 (PMC7542647; doi:10.1093/ntr/ntaa066)
Supplement: ntaa066_suppl_Supplementary_Taxonomy_Form [file ntaa066_suppl_supplementary_taxonomy_form.pdf]

NICOTINE & TOBACCO RESEARCH

SRNT

Contributorship form for publication in *Nicotine & Tobacco Research*

Authorship Contribution

In submitting this manuscript to *Nicotine & Tobacco Research*, the corresponding author and any co-authors are confirming that each and every author has, in accordance with the ICMJE guidelines, (a) made a substantial contribution to the conception and design, acquisition of data, and/or analysis and interpretation of data, (b) participated in the drafting of the article or revising it critically for important intellectual content, (c) have read and approved the final manuscript, AND (d) that the manuscript is not, either in whole or in part, currently under consideration by any other scientific journal and has not been previously published in either hard copy or electronic format. Except for those submitting the manuscript, members of Study Groups should not be individually cited as authors; instead the Study Group should be named as an author, and individuals within the group listed in full in an acknowledgement section of the manuscript.

This journal also requires that each and every author's specific contributions to the manuscript be described using the Contributor Roles Taxonomy (CRediT). This taxonomy includes 14 descriptors representing the roles typically played by contributors to scientific scholarly outputs, and the roles describe each contributor's specific contributions to the manuscript. **For accepted manuscripts, this completed form will publish online as supplementary material.**

Required Information

Manuscript Reference (e.g. NTR-2019-0378.R2): **NTR-2020-096**

Manuscript Title: **Pregnant Smokers Receiving Opioid Agonist Therapy Have an Elevated Nicotine Metabolite Ratio: A Replication Study**

Corresponding Author: Henry R. Kranzler

Number of Authors: 8

A detailed explanation defining the contribution of each author to the manuscript must be made using the table below.

Corresponding Author

As the corresponding author by signing this authorship form you confirm on behalf of yourself and all authors on the paper that this manuscript complies with the authorship policies of *Nicotine & Tobacco Research*.

Full First and Surname: **Henry Kranzler**

Signature: Henry R. Kranzler, MD **Date: March 27, 2020**

Declaration

The corresponding author must state the contribution of *all authors* to the manuscript. If there are more than 20 co-authors, please add additional rows to the table below.

	Author Name	Conceptualization	Data Curation	Formal Analysis	Funding Acquisition	Investigation	Methodology	Project Administration	Resources	Software	Supervision	Validation	Visualization	Writing – Original Draft	Writing – Review & Editing
1	Henry Kranzler	☒	☒	☐	☒	☒	☒	☒	☒	☐	☒	☐	☐	☒	☒
2	Yukiko Washio	☐	☐	☐	☐	☒	☐	☒	☐	☐	☒	☐	☐	☐	☒
3	Leah Zindel	☐	☒	☐	☐	☒	☐	☒	☐	☐	☐	☐	☐	☐	☒
4	Kevin Lynch	☒	☒	☒	☐	☐	☐	☐	☐	☐	☐	☐	☐	☒	☒
5	Dennis Hand	☐	☐	☐	☐	☒	☐	☒	☐	☐	☒	☐	☐	☐	☒
6	Rachel Tyndale	☐	☒	☒	☒	☒	☐	☐	☐	☐	☒	☐	☐	☐	☒
7	Robert Schnoll	☒	☐	☐	☒	☒	☒	☐	☐	☐	☒	☐	☐	☐	☒
8	Cheryl Oncken	☒	☐	☐	☒	☐	☒	☐	☐	☐	☐	☐	☐	☐	☒
9	Click here to enter text.	☐	☐	☐	☐	☐	☐	☐	☐	☐	☐	☐	☐	☐	☐
10	Click here to enter text.	☐	☐	☐	☐	☐	☐	☐	☐	☐	☐	☐	☐	☐	☐
11	Click here to enter text.	☐	☐	☐	☐	☐	☐	☐	☐	☐	☐	☐	☐	☐	☐
12	Click here to enter text.	☐	☐	☐	☐	☐	☐	☐	☐	☐	☐	☐	☐	☐	☐
13	Click here to enter text.	☐	☐	☐	☐	☐	☐	☐	☐	☐	☐	☐	☐	☐	☐
14	Click here to enter text.	☐	☐	☐	☐	☐	☐	☐	☐	☐	☐	☐	☐	☐	☐
15	Click here to enter text.	☐	☐	☐	☐	☐	☐	☐	☐	☐	☐	☐	☐	☐	☐
16	Click here to enter text.	☐	☐	☐	☐	☐	☐	☐	☐	☐	☐	☐	☐	☐	☐
17	Click here to enter text.	☐	☐	☐	☐	☐	☐	☐	☐	☐	☐	☐	☐	☐	☐
18	Click here to enter text.	☐	☐	☐	☐	☐	☐	☐	☐	☐	☐	☐	☐	☐	☐
19	Click here to enter text.	☐	☐	☐	☐	☐	☐	☐	☐	☐	☐	☐	☐	☐	☐
20	Click here to enter text.	☐	☐	☐	☐	☐	☐	☐	☐	☐	☐	☐	☐	☐	☐
